# Insulin Encapsulation in a Chitosan–Alginate Matrix and In Vitro Release

**DOI:** 10.3390/bioengineering13070812

**Published:** 2026-07-16

**Authors:** Ruth Reyes, María Luisa Ojeda-Martínez, Armin Hernández-Gordillo, Miguel Ojeda-Martínez, Sonia Sifuentes-Franco, Mario E. Cano, Mariana Díaz-Zaragoza, Celso Velásquez-Ordóñez

**Affiliations:** 1 Departamento de Ciencias Naturales y Exactas, Centro Universitario de los Valles, Universidad de Guadalajara, Ameca 46600, Jalisco, Mexico; ruth.reyes@academicos.udg.mx (R.R.); maria.ojeda@academicos.udg.mx (M.L.O.-M.); armin.hernandez@academicos.udg.mx (A.H.-G.); miguel.ojeda9380@academicos.udg.mx (M.O.-M.); 2 Departamento de Ciencias de la Salud, Centro Universitario de los Valles, Universidad de Guadalajara, Ameca 46600, Jalisco, Mexico; sonia.sifuentes@academicos.udg.mx (S.S.-F.); mariana.diaz@academicos.udg.mx (M.D.-Z.); 3 Division de Desarrollo Biotecnologico, Centro Universitario de la Cienega, Universidad de Guadalajara, Av. Universidad, 1115, Ocotlan 47820, Jalisco, Mexico; mario.cano@academicos.udg.mx

**Keywords:** insulin encapsulation, chitosan–alginate nanoparticles, biomaterials

## Abstract

**Background/Objectives**: Although insulin therapy has been fundamental in the management of diabetes mellitus since its discovery, limitations associated with conventional administration routes continue to drive the development of alternative delivery strategies. This study reports the design, synthesis, and characterization of a chitosan–alginate nanoparticulate system for the encapsulation and pH-responsive release behavior of recombinant human insulin, developed via ionic crosslinking with sodium tripolyphosphate (TPP). **Methods**: Nanoparticles were prepared by ionic crosslinking. Physicochemical characterization was carried out by UV–vis spectroscopy, Fourier-transform infrared spectroscopy with attenuated total reflectance (FTIR–ATR), dynamic light scattering (DLS), zeta potential analysis, fluorescence spectroscopy, and scanning electron microscopy (SEM). In vitro release studies were conducted at pH 4.5 and 7.4 to simulate physiological environments. **Results**: The nanoparticles achieved an encapsulation efficiency (EE%) of 30 ± 2.6% and a zeta potential of 41 ± 1.7 mV (pH 4.0). SEM analysis revealed mostly elongated nanoparticles, while DLS measurements confirmed nanometric sizes. The release profile demonstrated rapid insulin release at acidic pH (4.5) and sustained release at slightly basic pH (7.4). After three months of storage at room temperature, the lyophilized nanoparticles allowed continued release at pH 7.4. **Conclusions**: The synthesized chitosan–alginate nanoparticles provide a biocompatible platform for the encapsulation and release of recombinant human insulin. These findings can contribute to the development of nanosystems as an alternative for insulin delivery, thereby improving therapeutic adherence and accessibility.

## 1. Introduction

Since its discovery in 1922, insulin has remained an important component of diabetes management, with evolving pharmaceutical formulations proving essential in enhancing the survival and quality of life of patients with both type 1 (T1DM) and type 2 (T2DM) diabetes mellitus [1]. Diabetes mellitus is a chronic metabolic disorder characterized by hyperglycemia caused by insufficient insulin secretion or action, or both [2]. Globally, diabetes continues to be a leading cause of morbidity and mortality, with its prevalence rising alarmingly [3]. According to the International Diabetes Federation, approximately 588.7 million adults lived with diabetes in 2024, and this number is projected to reach 852.5 million by 2050 [4]. The three countries with the highest number of type 1 diabetes cases in 2025 are the USA with 1,513,000 people, India with 987,000 people, and China with 626,000 people [5].

Although insulin formulations have proven effective, treatment adherence remains a challenge because current administration routes are invasive and dosing is frequent. Subcutaneous injection is the standard method for insulin administration; however, it is associated with local pain, discomfort, risk of infection, and social stigma, all of which significantly impact treatment adherence and quality of life. Furthermore, self-medication is quite common among diabetes patients, and unsupervised insulin dose adjustments can lead to severe hypoglycemia or even fatal overdose [6].

Oral or transdermal administration represents a more physiological and convenient approach for the patient. Unfortunately, the oral bioavailability of insulin is very low due to enzymatic degradation in the gastrointestinal tract and its poor permeability across intestinal epithelial barriers [7,8,9].

To address these limitations, nanotechnology has emerged as a promising tool for developing advanced nanosystems for drug delivery that could protect sensitive biomolecules and modulate their release kinetics [10].

Among polymeric nanocarriers, chitosan (CS) and alginate (ALG) are useful due to their biocompatibility, biodegradability, and ability to form polyelectrolyte complexes through ionic interactions. Chitosan, a cationic polysaccharide derived from chitin, has mucoadhesive and permeable properties that facilitate drug absorption across biological membranes. Alginate, an anionic polysaccharide extracted from brown algae, provides excellent gelling capacity in physiological environments [11,12,13,14,15].

The combination of chitosan (CS) and alginate (ALG) allows the synthesis of polyelectrolytic complexes with adjustable physicochemical properties that are ideal for encapsulating biomolecules such as insulin. The ionic crosslinking technique using sodium tripolyphosphate (TPP) as the crosslinking agent enables non-aggressive aqueous processing without the need for organic solvents or high temperatures, aiming to minimize the loss of bioactivity of the encapsulated protein [16,17,18,19,20]. Previous studies have demonstrated the potential of chitosan–alginate nanoparticles as carriers for proteins, peptides, and other macromolecules. However, optimizing formulation parameters such as polymer ratio and pH remains important for achieving optimal release [21,22,23,24,25,26].

The present work focuses on the synthesis and characterization of chitosan–alginate nanoparticles that encapsulate recombinant human insulin via ionic crosslinking. The study aims to evaluate their physicochemical characteristics and analyze encapsulation efficiency and release kinetics at different pH values.

## 2. Materials and Methods

### 2.1. Materials

Recombinant human insulin (Sigma-Aldrich, Saint Louis, MO, USA) was used as a model protein. Low molecular weight chitosan (CS), sodium alginate (ALG), and sodium tripolyphosphate (TPP) were obtained from Sigma-Aldrich. Acetic acid (glacial, 99.7%) and sodium hydroxide were of analytical grade. All solutions were prepared with triple-distilled water.

### 2.2. Preparation of Chitosan–Alginate Nanoparticles Loaded with Insulin

#### 2.2.1. Preparation of Base Solutions

The stock solutions used for nanoparticle synthesis were prepared as follows:

- S1-CS: Chitosan solution at 2.5 mg/mL, dissolved in 0.4 M acetic acid previously adjusted to pH 3.0.
- S2-TPP: Sodium tripolyphosphate at 0.5 mg/mL, prepared in 0.01 M NaOH.
- S3-ALG: Sodium alginate at 1.5 mg/mL, dissolved in 0.01 M NaOH.
- S4-INS: Recombinant human insulin at 2 mg/mL, dissolved in 0.01 M HCl.

#### 2.2.2. Synthesis of Chitosan–Alginate Nanoparticles

Nanoparticles were synthesized through the ionic crosslinking method, following the procedure described by Calvo et al. [17], with slight modifications and controlled pH conditions. The S2-TPP and S3-ALG solutions were first mixed to form a single phase, which was then added dropwise to the S1-CS solution using a syringe pump at a constant flow rate of 0.13 mL/min for 30 min under continuous magnetic stirring.

#### 2.2.3. Encapsulation of Recombinant Human Insulin

For insulin encapsulation, 2 mL of S4-INS was mixed with 1 mL of S2-TPP and 1 mL of S3-ALG. The mixture was added dropwise to 4 mL of S1-CS using a syringe pump (0.13 mL/min) under constant stirring. This step promoted ionic crosslinking and facilitated the efficient incorporation of insulin into the chitosan–alginate matrix. The resulting suspension was allowed to equilibrate for 30 min at room temperature before further characterization.

#### 2.2.4. pH Monitoring

The pH of all individual solutions and intermediate mixtures was continuously monitored throughout the synthesis process to ensure optimal ionic crosslinking and encapsulation conditions. pH adjustments were made using 0.1 M NaOH or 0.1 M HCl when necessary.

### 2.3. Determination of Encapsulation Efficiency (EE%) and Loading Capacity (LC%)

The encapsulation efficiency was determined from the difference between the total insulin used and the amount of free insulin remaining in the supernatant. Quantification was performed by UV–Vis spectrophotometry (Cary 300 Varian, MULGRAVE Victoria, Australia), with insulin absorbance measured at its characteristic wavelength. The EE% was calculated using Equation (1) [27]:

(1) $$EE\%=\frac{\left(C_{i}-C_{f}\right)}{C_{i}}\times 100,$$

where $C_{i}$ is the initial insulin concentration and $C_{f}$ is the concentration in the supernatant liquid.

The loading capacity was estimated from thermogravimetric analysis (TGA) by comparing the mass loss in the polymer degradation region of blank and insulin-loaded nanoparticles.

### 2.4. Characterization of Nanoparticles

#### 2.4.1. UV–Vis Spectroscopy

UV–Vis spectra were recorded using a Cary 300 spectrophotometer (Varian, MULGRAVE Victoria, Australia) in the range of 200–350 nm with quartz cuvettes. Measurements were performed at room temperature, except for in vitro release studies, where samples were maintained at 36–37 °C to simulate physiological conditions.

#### 2.4.2. Dynamic Light Scattering (DLS) and Zeta Potential

Particle size and zeta potential were determined using a Zetasizer Nano ZS90 (Malvern Instruments). Measurements were taken at 25 °C in a quartz cuvette with water as a dispersant (refractive index = 1.330, viscosity = 0.8872 cP). Each measurement lasted 60 s, with a detection position of 4.65 mm and an attenuator level of 10. The material’s refractive index was set to 1.60 with an absorption value of 0.100. Zeta potential (ξ) was measured using the Zeta Dip Cell under an applied electric field between electrodes.

#### 2.4.3. Lyophilization

Following encapsulation, nanoparticle suspensions were frozen at −46 °C for 24 h and subsequently lyophilized using a BioBase tabletop freeze-dryer (Shandong, China) for another 24 h. The resulting powders were collected and stored at room temperature as dry formulations.

#### 2.4.4. FTIR–ATR Spectroscopy

FTIR spectra were acquired using a Varian 600 spectrometer (MULGRAVE Victoria, Australia) equipped with an ATR accessory in the range of 4000–500 cm^−1^, with 32 scans at 4 cm^−1^ resolution under ambient conditions.

#### 2.4.5. Fluorescence Spectroscopy

Fluorescence spectra were obtained using a Varian Eclipse spectrofluorometer (MULGRAVE Victoria, Australia) at an excitation wavelength of 275 nm. Measurements were performed in the liquid state at room temperature, except for in vitro release assays, where samples were maintained at 36–37 °C.

#### 2.4.6. Scanning Electron Microscopy (SEM)

The morphology of the nanoparticles was observed using a JEOL JSM-6610LV scanning electron microscope (Tokyo, Japan). The samples were analyzed in colloidal form by depositing a drop of the nanoparticle suspension onto carbon tape and allowing it to dry at room temperature prior to imaging. Once dried, the samples were sputter-coated with gold for 20 s to improve the conductivity. Images were obtained in high-vacuum mode at 5 kV, 18.8 °C, and 30.4% relative humidity.

#### 2.4.7. Thermogravimetric Analysis (TGA)

Thermogravimetric analysis (TGA) was performed to evaluate the thermal stability of the materials and to provide complementary evidence of insulin incorporation into the chitosan–alginate nanoparticles. The samples were deposited onto a platinum sample holder of a thermogravimetric analyzer (TGA 1000, ISI, Boerne, TX, USA). The analysis was conducted in an inert nitrogen (N_2_) atmosphere to prevent oxidative degradation. A constant heating rate of 10 °C·min^−1^ was applied, and the mass loss was recorded as a function of temperature.

### 2.5. In Vitro Release Studies

In vitro release of insulin was evaluated under simulated physiological conditions (36–37 °C) using 5 mL of phosphate buffer media at pH 4.5 and 7.4 under constant agitation at 350 rpm to assess the effect of pH on release kinetics. Simulated gastrointestinal conditions were also evaluated using media at pH 1.2, 4.5, 6.8, and 7.4, adjusted with HCl or NaOH solutions as required.

#### 2.5.1. UV–Vis Monitoring

Release assays were monitored by UV–Vis spectrophotometry (Cary 300, Varian) over the range of 200–350 nm. Absorbance readings were recorded every 5 min during the first 30 min of release.

#### 2.5.2. Fluorescence Monitoring

Fluorescence measurements were performed under the same conditions as the UV–Vis release assays, using an excitation wavelength of 275 nm and monitoring emission in the 275–400 nm range, with readings taken every 5 min during the initial 30 min of release.

#### 2.5.3. Drug Release Kinetics

The Higuchi model was constructed by plotting the cumulative percentage of drug released versus the square root of time.

The Korsmeyer–Peppas model was applied by plotting the logarithm of cumulative percentage drug release versus the logarithm of time.

## 3. Results

### 3.1. pH Monitoring

The pH was continuously monitored throughout each stage of nanoparticle synthesis (Table 1). Maintaining controlled conditions during the ionic crosslinking process was essential to prevent insulin precipitation or denaturation. The final colloidal suspension remained transparent at pH 4.0, slightly above the pKa of alginate (~3.5), indicating that a significant fraction of carboxyl groups were deprotonated (–COO^−^). This environment allowed the solubility and compatibility of all precursors, ensuring the electrostatic interactions between the amino groups of chitosan (–NH_3_^+^) and the carboxylate groups of alginate (–COO^−^), benefiting the formation of nanoparticles without degrading the protein.

### 3.2. UV–Vis Characterization

**Figure 1:**
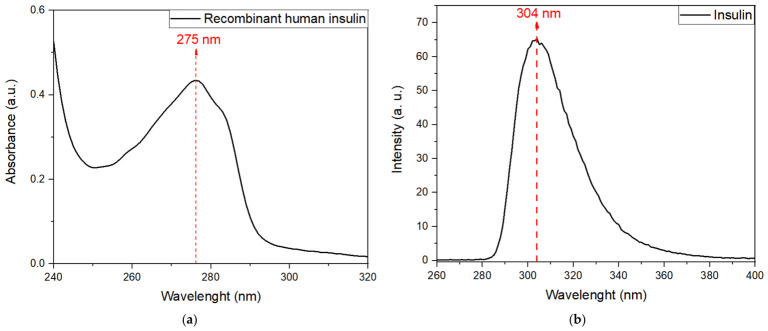
(**a**) UV–Vis and (**b**) fluorescence spectra of recombinant human insulin in triple-distilled water. The UV–Vis spectrum shows the characteristic absorption peak at 275 nm, while the fluorescence spectrum was obtained after excitation at 275 nm.

The UV–Vis spectrum of recombinant human insulin exhibited a maximum absorption peak at 275 nm, corresponding to Figure 1a, which is characteristic of the electronic transitions of aromatic amino acids such as tyrosine and phenylalanine. This absorption band confirms the presence of insulin in the aqueous solution. This wavelength was used to quantify unencapsulated insulin in the supernatant and to construct the calibration curve for determining encapsulation efficiency and release profiles.

### 3.3. Physical Characterization

The main physicochemical parameters of the chitosan–alginate nanoparticles are summarized in Table 2, including particle size distribution (DLS), zeta potential (ξ), polydispersity index (PDI), and encapsulation efficiency (EE%). Measurements were performed for both blank and insulin-loaded nanoparticles (0.25 mg/mL) after two days of storage at 4 °C to evaluate short-term colloidal stability.

Blank nanoparticles exhibited an average hydrodynamic diameter of 688 ± 54 nm by intensity (82 ± 15 nm by number) with a PDI of 0.53 ± 0.02 and a zeta potential of 27.5 ± 2.2 mV, indicating moderate colloidal stability.

Upon insulin loading, nanoparticles showed a slightly larger average size (663 ± 33 nm by intensity; 101 ± 20 nm by number) and a lower PDI (0.28 ± 0.02); although the difference between intensity-weighted and number-weighted distributions indicates the presence of particle heterogeneity, the reduced PDI suggests a narrower size distribution compared with blank nanoparticles. The increase in zeta potential to 41 ± 1.7 mV reflects enhanced surface charge repulsion, which contributes to improved suspension stability.

The encapsulation efficiency (EE% = 30 ± 2.6) confirmed successful insulin loading within the chitosan–alginate matrix; this result is consistent with electrostatic interactions between the protonated amine groups of chitosan and the negatively charged insulin molecules.

The loading capacity (LC% = 6.7 ± 0.3%) was estimated from thermogravimetric analysis as the apparent contribution of insulin to the overall mass loss of the system. This parameter reflects the relative amount of insulin effectively associated with the nanoparticles.

To evaluate the physical stability of the formulation, the physicochemical properties of insulin-loaded chitosan–alginate nanoparticles were re-assessed after one month of storage. The mean hydrodynamic diameter remained at 530 ± 4.7 nm, indicating excellent reproducibility and minimal size variation over time. The polydispersity index (PDI) was still low (0.26 ± 0.02). The zeta potential remained highly positive at 41 ± 0.3 mV, indicating strong electrostatic repulsion between particles.

### 3.4. FTIR–ATR Characterization

**Figure 2:**
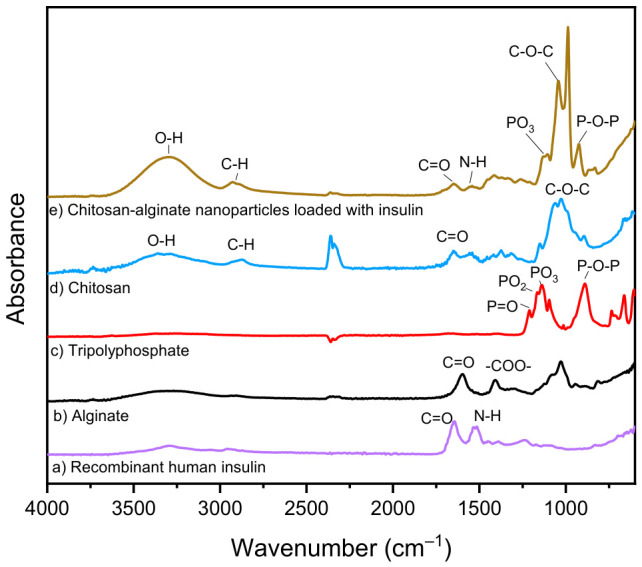
FTIR spectra of (**a**) Recombinant human insulin; (**b**) Alginate; (**c**) Tripolyphosphate; (**d**) Chitosan; (**e**) Chitosan−alginate nanoparticles loaded with insulin.

FTIR–ATR spectroscopy was employed to confirm the chemical interactions between chitosan, alginate, and insulin within the nanoparticle matrix. Figure 2 presents the characteristic spectra of pure components (chitosan, alginate and insulin) and the insulin-loaded chitosan–alginate nanoparticles.

For pure insulin (Figure 2a), characteristic bands were observed at 1592 cm^−1^, associated with the C=O stretching of amide groups, and at 1479 cm^−1^, corresponding to N–H bending, confirming the presence of amide and amine functional groups typical of protein structures. The alginate spectrum (Figure 2b) showed strong absorption at 1596 cm^−1^ (C=O) and 1405 cm^−1^ (COO^−^), confirming the carboxylate groups. Chitosan (Figure 2d) exhibited broad O–H and N–H stretching around 3343–3261 cm^−1^, C–H stretching at 2875 cm^−1^, and amide bands at 1646 and 1560 cm^−1^, together with a C–O–C peak at 1011 cm^−1^. The TPP spectrum (Figure 2c) displayed phosphate-related bands at 1213 cm^−1^ (P=O), 1168 cm^−1^ (PO_2_), 1133 cm^−1^ (PO_3_), and 887 cm^−1^ (P–O–P), confirming its structure [28,29,30,31].

After nanoparticle formation (Figure 2e), the FTIR spectrum exhibited combined and shifted peaks indicative of molecular interactions among insulin, chitosan, alginate, and TPP. The appearance of bands at 3234 cm^−1^ (O–H), 2879 cm^−1^ (C–H), 1594 cm^−1^ (C=O), and 1490 cm^−1^ (N–H) confirmed successful encapsulation. In addition, new bands at 1083 cm^−1^ (PO_3_), 993 cm^−1^ (C–O–C), and 875 cm^−1^ (P–O–P) evidenced phosphate crosslinking between the polymers and TPP. In lyophilized nanoparticles, the persistence of characteristic bands at 1598, 1490, and 1205 cm^−1^, corresponding to C=O, N–H, and C–N stretching, confirmed the presence and structural integrity of insulin after freeze-drying.

### 3.5. Fluorescence Analysis

Fluorescence spectroscopy was used to monitor insulin’s conformational integrity. Insulin emitted a fluorescence peak at 304 nm upon excitation at 275 nm (Figure 1b), corresponding to aromatic amino acid residues, including tryptophan, tyrosine, and phenylalanine [32]. The presence of this emission peak confirmed that the protein maintained its native tertiary structure after encapsulation, with no evidence of denaturation.

### 3.6. Morphological Characterization

**Figure 3:**
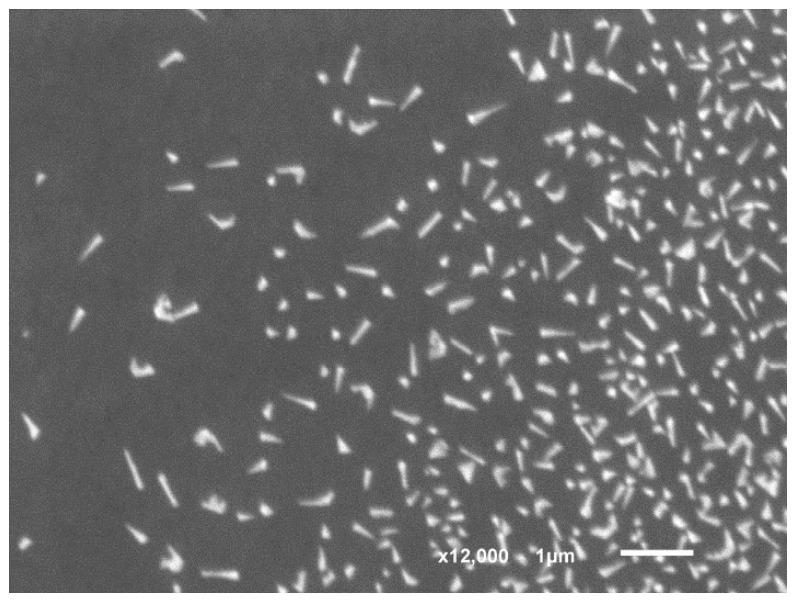
SEM micrograph of chitosan–alginate nanoparticles with encapsulated insulin at a magnification of 12,000×.

The morphology of the chitosan–alginate nanoparticles encapsulating insulin was analyzed by SEM at a magnification of 12,000× (Figure 3). The micrograph revealed a heterogeneous distribution with predominantly elongated particles, along with smaller spherical structures that may represent frontal projections of cylindrical nanoparticles or minor agglomerates.

**Figure 4:**
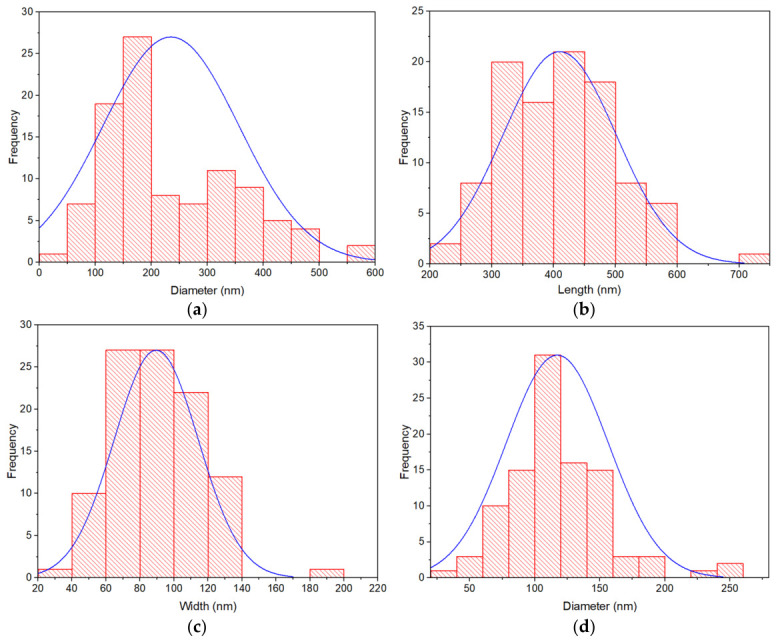
(**a**) Diameter distribution of chitosan–alginate nanoparticles with encapsulated insulin; (**b**) Length distribution of elongated chitosan–alginate nanoparticles with encapsulated insulin; (**c**) Width distribution of elongated chitosan–alginate nanoparticles with encapsulated insulin; (**d**) Diameter distribution of spherical chitosan–alginate nanoparticles with encapsulated insulin.

Size distribution histograms (Figure 4), analyzed using Feret’s diameter measurements, showed that most particles exhibited diameters below 250 nm, with the highest frequency between 100 and 200 nm. Elongated nanoparticles displayed lengths mainly between 300 and 500 nm (modal ≈ 430 nm) and widths between 70 and 100 nm. Spherical nanoparticles had diameters concentrated between 90 and 130 nm (modal ≈ 110 nm).

This dimensional range confirms that the nanoparticles are within the optimal size for cellular uptake and drug delivery through biological barriers.

### 3.7. In Vitro Release Studies

**Figure 5:**
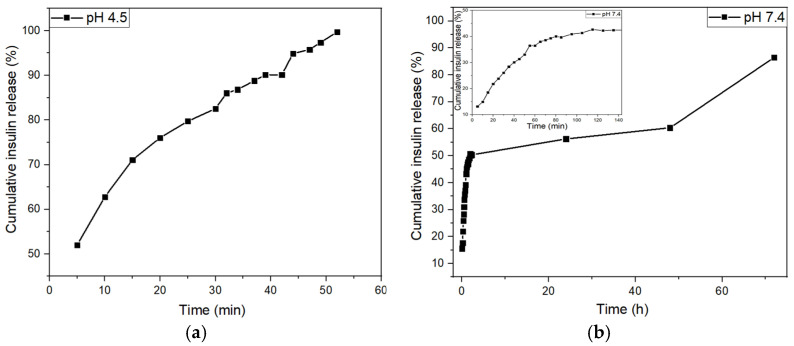
Cumulative insulin release percentages: (**a**) pH 4.5; (**b**) pH 7.4.

**Figure 6:**
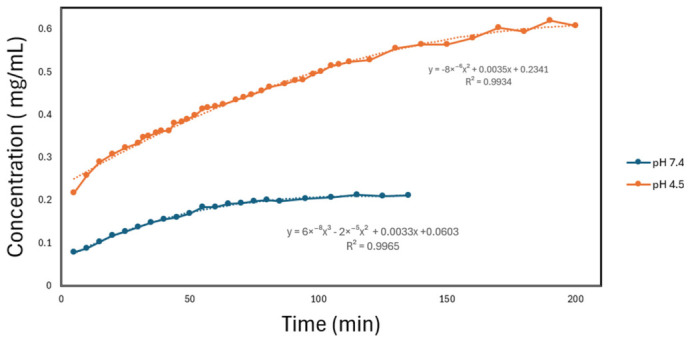
Concentration (mg/mL) vs. monitoring time in UV–Vis at pH 4.5 and 7.4.

**Figure 7:**
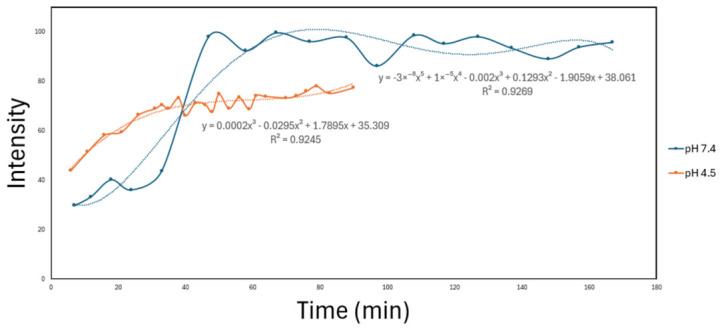
Fluorescence intensity vs. monitoring time at pH 4.5 and 7.4.

**Figure 8:**
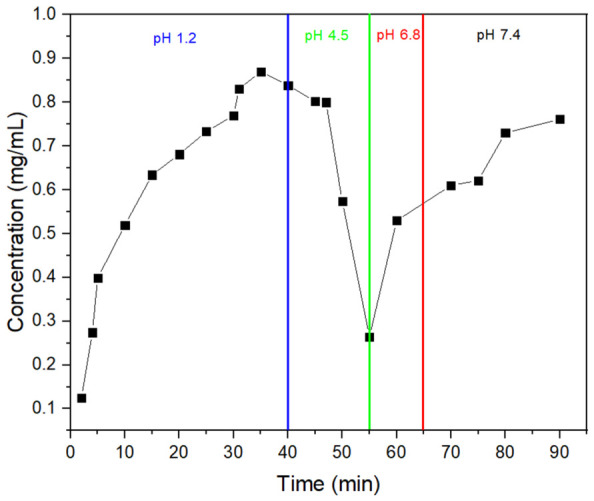
Concentration (mg/mL) vs. UV–Vis release time of insulin in a simulated gastrointestinal tract.

The release profiles of insulin-loaded nanoparticles at pH 4.5 and 7.4 are shown in Figure 5. At pH 4.5, the insulin release exhibited a second-degree polynomial fit under UV–vis monitoring and a third-degree fit under fluorescence monitoring (Figure 6 and Figure 7), indicating rapid desorption during the first 30 min (52.0% at 5 min; 82.1% at 30 min) and nearly complete release (99.7%) within 52 min. The faster release in acidic medium can be attributed to enhanced protonation and swelling of chitosan, which lead to partial disintegration of the nanoparticles and facilitate insulin diffusion through the polymer matrix. At pH 7.4, the insulin release exhibited a third-order polynomial profile in UV–vis monitoring and a sixth-degree polynomial trend in fluorescence monitoring, as illustrated in Figure 7 and Figure 8, showing an initial burst (15.57% at 5 min) followed by a gradual release (56.25% at 24 h; 86.46% at 72 h). The lower solubility of chitosan at neutral pH restricts insulin diffusion, producing a sustained release behavior suitable for transdermal environments.

UV–Vis and fluorescence monitoring (Figure 6 and Figure 7) confirmed these trends: the emission intensity increased rapidly at pH 4.5, whereas a delayed and complex response was observed at pH 7.4, suggesting a multi-phase release mechanism.

The cumulative insulin release profiles from chitosan–alginate nanoparticles were analyzed using Higuchi and Korsmeyer–Peppas kinetic models.

At pH 4.5, the Higuchi model exhibited the highest coefficient of determination (R^2^ = 0.9953) with a diffusion constant (kH = 7.8055), followed by the Korsmeyer–Peppas model (R^2^ = 0.9907, *n* = 0.2895). At pH 7.4, the Higuchi model also showed the best fit (R^2^ = 0.9413, kH = 3.4555), while the Korsmeyer–Peppas model yielded R^2^ = 0.9725 and *n* = 0.4019.

The kinetic parameters obtained from both models are summarized in Table 3.

### 3.8. Simulated Gastrointestinal Release

To evaluate the potential oral delivery behavior, nanoparticles were exposed to sequential pH changes (1.2 → 4.5 → 6.8 → 7.4) mimicking gastrointestinal transit. Figure 8 shows that under gastric conditions (pH 1.2), a rapid initial release was observed, reaching a concentration of 0.87 mg/mL within the first 35 min. Upon adjusting the pH to 4.5, the detectable insulin concentration experienced a sharp decline to 0.26 mg/mL at 55 min. However, as the pH was further increased to 7.4, the system showed a recovery phase, with concentration levels rising back to 0.76 mg/mL by the end of the 90 min study. This bimodal profile indicates a dynamic response to environmental changes.

### 3.9. Release Studies of Lyophilized Nanoparticles After Three Months of Storage

**Figure 9:**
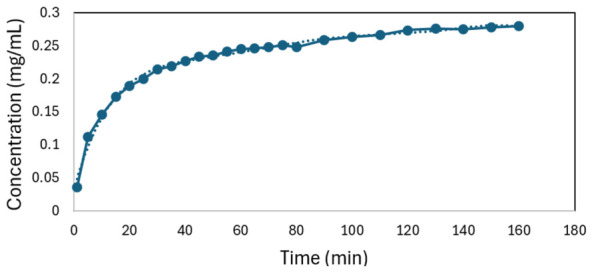
Concentration vs. UV–Vis monitoring time of insulin release encapsulated in chitosan–alginate nanoparticles at pH 7.4 after 3 months of storage at room temperature.

The release profile at pH 7.4 (Figure 9) fitted a sixth-order polynomial (R^2^ = 0.9921) by UV–Vis monitoring, suggesting slightly more complex release kinetics than the freshly prepared samples.

**Figure 10:**
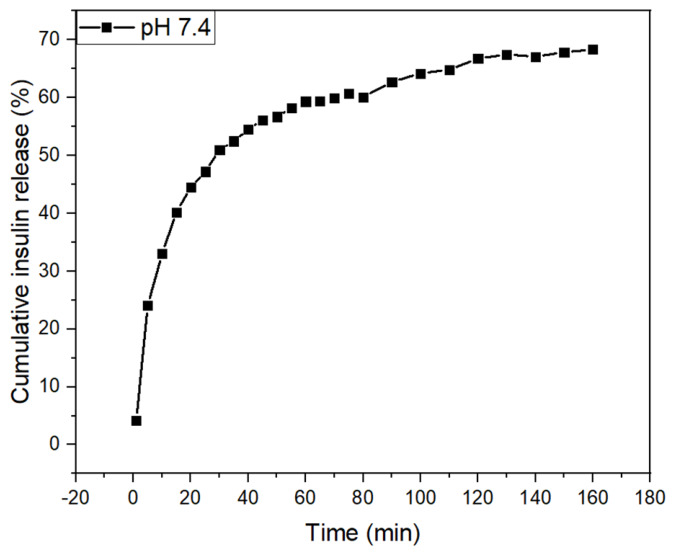
Cumulative release percentages at pH 7.4 of insulin encapsulated in chitosan−alginate nanoparticles after 3 months without refrigeration.

**Figure 11:**
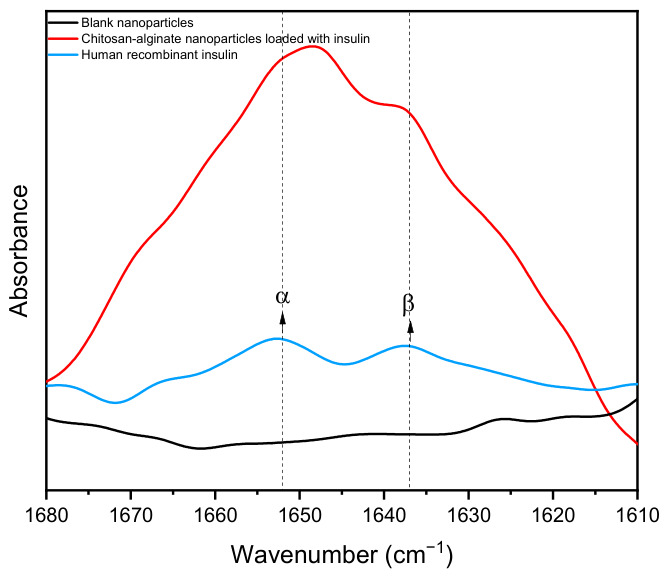
FTIR spectrogram of lyophilized chitosan−alginate nanoparticles without insulin, with insulin, and recombinant human insulin.

After three months of storage at room temperature, release tests of insulin encapsulated in chitosan–alginate nanoparticles were performed by UV–Vis characterization (Figure 10 and Figure 11).

Comparison between refrigerated (Figure 5b) and non-refrigerated (Figure 10) samples revealed minor differences in release rates: at 5 min, non-refrigerated nanoparticles released 24.07% versus 15.57% for refrigerated ones, and at 30 min, 50.96% versus 30.92%, respectively. Both systems exhibited comparable cumulative release behavior, demonstrating retention of release performance after three months of storage at room temperature, which is a key advantage for applications in regions with limited cold-chain infrastructure.

### 3.10. Amide I Region Analysis of Encapsulated Insulin Assessed by FTIR

Figure 11 shows the FTIR spectrum of recombinant human insulin (blue line), where two main contributions can be clearly identified in the amide I region. A band centered at approximately 1648 cm^−1^ is observed, which is characteristic of α-helix structures, while a second contribution near 1637 cm^−1^ is attributed to β-sheet content. These values fall within the typical range reported for proteins with well-defined secondary structures, where α-helices are commonly detected between 1648 and 1658 cm^−1^ and β-sheets between 1623 and 1643 cm^−1^ [33].

The FTIR spectrum of insulin–chitosan–alginate nanoparticles (red line) exhibits bands in the same spectral regions, indicating the presence of insulin within the nanoparticulate system. Although slight shifts in peak positions are observed compared to free insulin, the characteristic amide I bands remain distinguishable.

**Figure 12:**
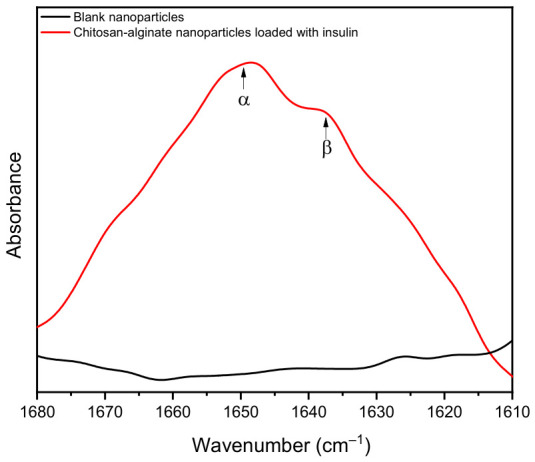
FTIR spectrogram of lyophilized chitosan−alginate nanoparticles without insulin and with insulin.

Figure 12 presents a comparison between lyophilized chitosan–alginate nanoparticles without insulin (black line) and insulin-loaded nanoparticles. The blank sample does not show significant absorption bands in the 1648 and 1637 cm^−1^ regions, confirming that these contributions arise from insulin rather than from the polymeric matrix.

### 3.11. Intrinsic Fluorescence Analysis of Aromatic Amino Acids of Encapsulated Insulin

**Figure 13:**
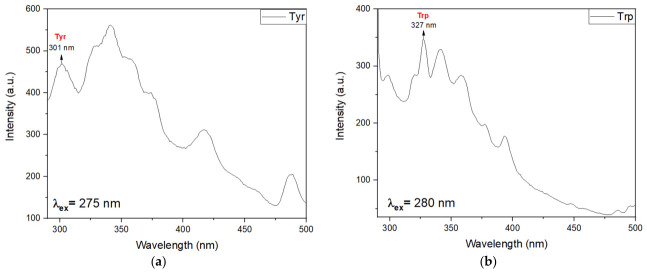
Fluorescence emission of (**a**) tyrosine in insulin-loaded chitosan–alginate nanoparticles after excitation at 275 nm, and (**b**) tryptophan in insulin-loaded chitosan–alginate nanoparticles after excitation at 280 nm.

Fluorescence spectroscopy was performed on encapsulated recombinant human insulin in order to evaluate the emission characteristics of its intrinsic aromatic amino acids. Figure 13a presents the fluorescence emission spectrum of insulin-loaded chitosan–alginate nanoparticles upon excitation at 275 nm, where the presence of tyrosine-related emissions confirms the presence of aromatic residues within the nanoparticulate system.

When the nanoparticles were excited at 280 nm, a dominant emission peak centered at 327 nm was observed (Figure 13b), corresponding to the typical fluorescence emission of tryptophan in an aqueous environment [34].

### 3.12. Hydrodynamic Size of Insulin Released from Chitosan–Alginate Nanoparticles

**Figure 14:**
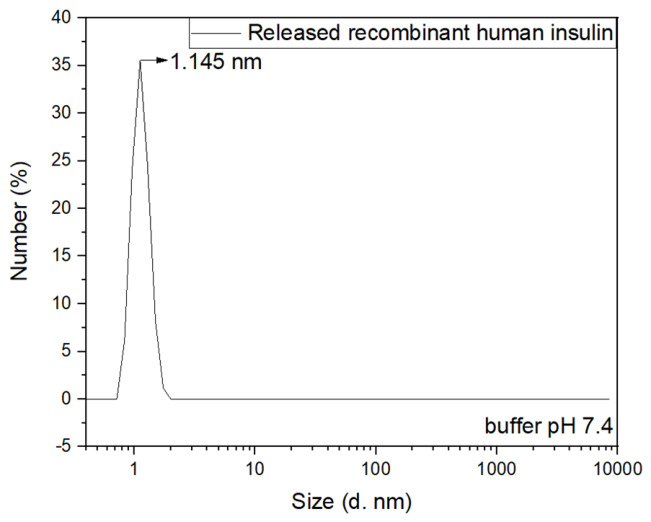
Size distribution by number of insulin-loaded chitosan−alginate nanoparticles.

Figure 14 shows the number-weighted size distribution of insulin released from chitosan–alginate nanoparticles after exposure to phosphate buffer (pH 7.4). Dynamic light scattering (DLS) analysis reveals a narrow and well-defined peak centered at approximately 1.145 nm.

The observed size distribution indicates the presence of a low-molecular-weight species in solution, consistent with released insulin. Notably, no additional peaks corresponding to larger particle sizes were detected, suggesting the absence of aggregates or residual nanoparticulate structures in the analyzed sample.

### 3.13. X-Ray Diffraction Analysis of Insulin-Loaded Chitosan–Alginate Nanoparticles

**Figure 15:**
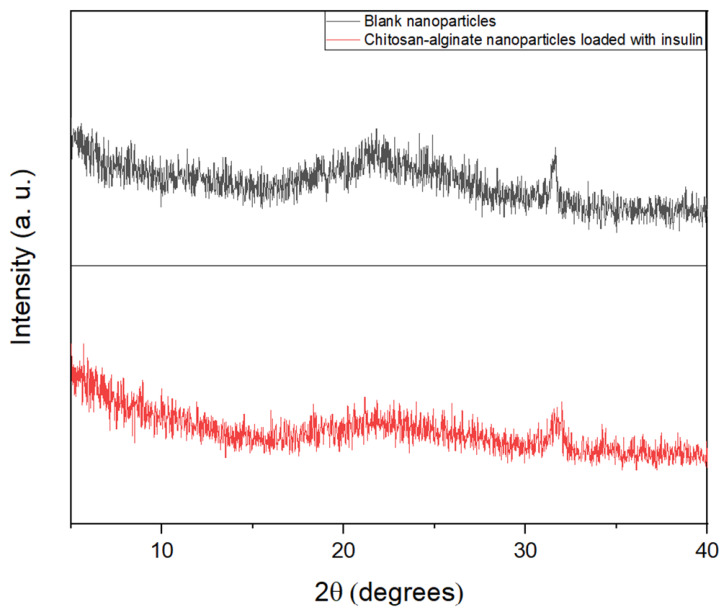
X-ray diffractograms of alginate–chitosan–TPP nanoparticles with and without insulin.

Figure 15 presents the X-ray diffraction (XRD) patterns of chitosan–alginate nanoparticles (blank) and nanoparticles loaded with recombinant human insulin. Both diffractograms exhibit a broad halo in the 2θ range between 10° and 30°, which is characteristic of a predominantly amorphous polymeric matrix formed by electrostatic interactions among alginate, chitosan, and tripolyphosphate (TPP) [35].

The XRD pattern of insulin-free nanoparticles shows no sharp diffraction peaks, confirming the amorphous nature of the chitosan–alginate–TPP network. Similarly, nanoparticles loaded with recombinant human insulin do not exhibit additional diffraction peaks attributed to crystalline insulin.

### 3.14. Thermogravimetric Analysis (TGA)

**Figure 16:**
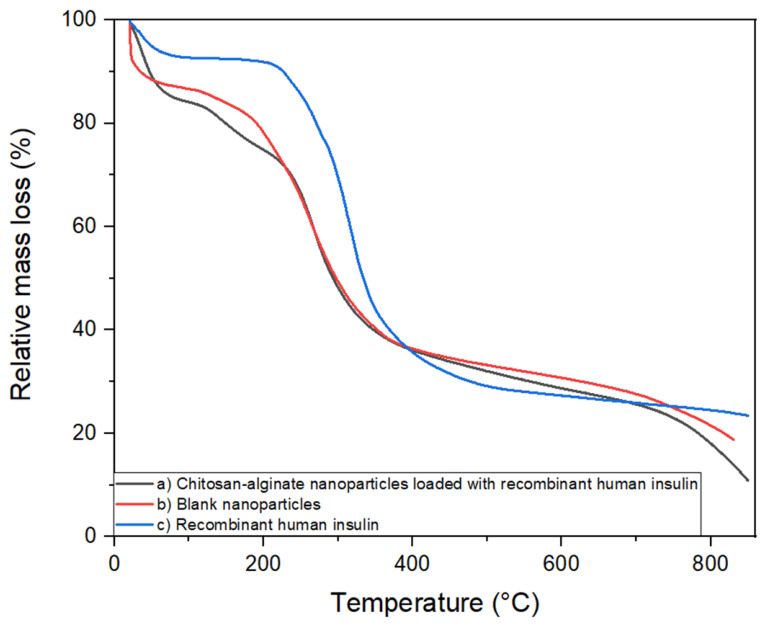
Thermogravimetric analysis (TGA) of chitosan–alginate nanoparticles with and without insulin compared to pure recombinant human insulin.

Figure 16 shows the thermogravimetric analysis (TGA) that was performed to evaluate the thermal stability and to provide indirect evidence of insulin incorporation within the chitosan–alginate nanoparticles.

**Figure 17:**
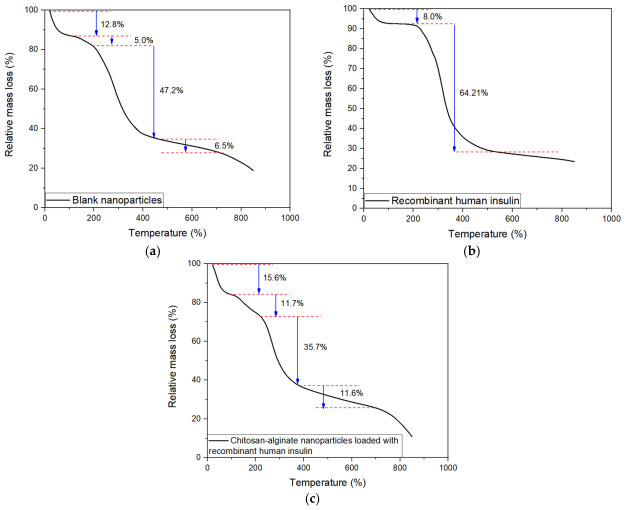
Stepwise mass loss analysis of thermogravimetric curves for (**a**) recombinant human insulin, (**b**) blank nanoparticles, and (**c**) chitosan–alginate nanoparticles loaded with human insulin.

All samples exhibited an initial weight loss below 150 °C, corresponding to the removal of physically adsorbed water. As shown in Figure 17, this mass loss was approximately 8.0% for pure insulin, 12.8% for blank nanoparticles, and 15.6% for insulin-loaded nanoparticles, indicating differences in hydration and structural organization.

The blank chitosan–alginate nanoparticles showed a major degradation step between 200 and 400 °C, with a mass loss of approximately 47.2%, which was attributed to the thermal decomposition of the polysaccharide matrix. In contrast, pure insulin exhibited sharper degradation (~64.2%) within a similar temperature range, consistent with protein denaturation and backbone degradation. The insulin-loaded nanoparticles displayed a distinct and broad degradation profile, with multiple steps (11.7%, 35.7%, and 11.6%), differing from both blank nanoparticles and pure insulin. This shift in degradation behavior indicates interactions between insulin and the polymeric matrix, suggesting successful incorporation. At higher temperatures (~800 °C), the residual mass of insulin-loaded nanoparticles was lower than that of blank nanoparticles, supporting the presence of an additional organic component (insulin) contributing to overall thermal decomposition. These results support the encapsulation of insulin within the chitosan–alginate system and confirm modifications in thermal behavior due to protein–polymer interactions.

## 4. Discussion

The results confirmed the successful design and synthesis of chitosan–alginate nanoparticles for encapsulating recombinant human insulin using ionic gelation. pH control during the synthesis process (Table 1) was important for maintaining protein stability. The final pH of 4.0 demonstrated protonation of the chitosan amino groups (–NH_3_^+^) and ionization of the alginate carboxyl groups (–COO^−^), promoting electrostatic complexation and nanoparticle formation without insulin denaturation or precipitation. The transparent appearance of the final colloidal suspension demonstrated adequate solubility and compatibility among all formulation components.

The UV–Vis spectrum of insulin showed its characteristic absorption at 275 nm, corresponding to the π→π* transitions of aromatic amino acids. This characteristic is suggestive of limited structural alteration of insulin and served as a reference for quantifying the unencapsulated fraction and for generating calibration curves.

Physicochemical parameters obtained from the DLS and zeta potential analyses demonstrated the efficacy of the formulation. Nanoparticles without insulin exhibited high polydispersity, while those loaded with insulin showed a lower polydispersity index (PDI) (0.28 ± 0.02), indicating a reduction in size dispersion after insulin incorporation. However, the difference between the intensity-weighted (663 ± 33 nm) and number-weighted (101 ± 20 nm) particle sizes suggests the presence of a heterogeneous particle population. This discrepancy is characteristic of DLS measurements, where a small fraction of larger particles or aggregates can disproportionately influence the intensity distribution. Therefore, the number-weighted distribution likely provides a more representative description of the predominant nanoparticle population [36,37].

The increase in surface charge from 27.5 ± 2.2 mV to 41 ± 1.7 mV suggests that insulin encapsulation increases the density of protonated sites on chitosan, contributing to colloidal stabilization. The encapsulation efficiency of 30 ± 2.6% is consistent with previous reports on ionic crosslinking (e.g., typical 25–35% in chitosan/insulin systems, Barbosa et al. [38] and Avadi et al. [39]), confirming successful electrostatic entrapment of insulin molecules. This moderate efficiency may be attributed to the hydrophilic nature of insulin, which favors its partitioning into the aqueous phase. Although higher encapsulation efficiencies would be desirable for therapeutic translation, the present formulation demonstrates successful insulin incorporation while maintaining favorable release behavior and structural characteristics. Future optimization of polymer ratios, crosslinking density, and insulin concentration may further improve loading performance.

Table 4 presents a comparison between the present formulation and previously reported polysaccharide-based insulin delivery systems.

Although chitosan- and alginate-based insulin carriers have been reported, fewer studies have evaluated chitosan–alginate systems prepared under mild aqueous conditions while simultaneously investigating structural integrity, pH-responsive release, lyophilized storage behavior, FTIR secondary structure analysis, intrinsic fluorescence, XRD, and thermogravimetric characterization.

After one month of storage, the mean hydrodynamic diameter remained at 529.2 ± 4.7 nm, indicating excellent reproducibility and minimal size variation over time. The polydispersity index (PDI) remained low (0.259 ± 0.0). The zeta potential remained highly positive at 41.1 ± 0.3 mV.

Spectroscopic analyses (FTIR–ATR) provided evidence of molecular interactions between the polymers, the crosslinker, and the protein. The shift and merging of characteristic amide, hydroxyl, and phosphate bands revealed the formation of ionic bridges and hydrogen bonds. These results support the formation of a CS–ALG–TPP matrix capable of protecting the insulin conformation, as further confirmed by fluorescence emission at 304 nm. The persistence of this emission band is consistent with preservation of characteristic structural features in the tertiary structure of insulin throughout encapsulation, validating the spectroscopic evidence obtained by FTIR.

Morphological evaluation by SEM revealed a mixture of elongated and spherical nanoparticles within the nanometric range (~100–500 nm). The size is optimal for epithelial uptake and endocytic transport, while the surface charge magnitude favors muco-adhesion and prolonged residence at biological interfaces. Although the appearance of elongated morphologies in chitosan–alginate systems is less frequently reported in the literature, some studies on chitosan/alginate have described non-spherical or rod-like particles (e.g., nanochitosan/alginate films; Vijayalakshmi et al. [42]) and particles of ~7.5 μm for alginate/chitosan systems for oral delivery of insulin (e.g., Zhang et al. [41]). We propose that, in our system, the elongated morphology may arise from anisotropic growth during ionic crosslinking under the chosen conditions and may contribute to enhanced interaction with mucosal tissues.

The release studies demonstrated pH-sensitive behavior. At acidic pH 4.5, rapid insulin release occurred due to protonation and swelling of chitosan, facilitating diffusion and partial matrix disintegration. At physiological pH 7.4, the reduced solubility of chitosan led to a slower, sustained release profile extending up to 72 h. This dual-phase behavior enables an initial burst for immediate therapeutic response followed by a prolonged maintenance phase of insulin levels.

The analysis and interpretation of the release kinetics were conducted following the approach described by Costa and Lobo [43] for modeling and comparison of dissolution profiles, which is widely used for evaluating the similarity and mechanistic behavior of release systems. The kinetic modeling suggests that insulin release from chitosan–alginate nanoparticles is predominantly governed by diffusion-controlled mechanisms under both evaluated pH conditions. The Higuchi model provided the best overall fit, suggesting that insulin release is predominantly governed by diffusion-controlled transport through the hydrated polymeric network. This behavior is typical of hydrogel-based delivery systems, where transport occurs through water-filled pores without significant contribution from polymer erosion within the studied time frame. This is further supported by the Korsmeyer–Peppas model, where the release exponent (*n* < 0.45) at both pH 4.5 (0.2895) and pH 7.4 (0.4019) confirms a Fickian diffusion mechanism.

The simulated gastrointestinal assay further confirmed the system’s responsiveness to ambient pH transitions. The initial release at pH 1.2 suggests that a significant fraction of insulin is released through the surface pores of the contracted matrix. The subsequent drop in concentration between 40 and 60 min is attributed to the approximation of the medium’s pH to the isoelectric point of human insulin during the addition of NaOH. This proximity likely induced a transient reduction in insulin solubility or protein aggregation, leading to lower absorbance readings. The subsequent recovery of concentration at pH 6.8–7.4 demonstrates the pH-responsiveness of the nanoparticles. The ionization of the polymer chains at neutral pH promotes matrix swelling, allowing the release of the remaining insulin core and the re-solubilization of the protein.

Release studies performed after three months of storage at room temperature showed that the nanoparticles retained a release profile comparable to that of freshly prepared samples.

The presence of well-defined amide I bands corresponding to α-helix and β-sheet structures in both free insulin and insulin-loaded nanoparticles suggests limited structural alteration of the secondary structure of insulin after encapsulation. The slight shifts observed in the insulin–chitosan–alginate system can be attributed to changes in the local chemical environment of the carbonyl groups, likely due to hydrogen bonding or electrostatic interactions between insulin and the polymeric matrix. Importantly, the absence of overlapping bands from the blank nanoparticles in the same spectral region supports the conclusion that the detected amide I signals originate exclusively from insulin. This observation confirms the successful incorporation of the protein into the nanoparticulate system and indicates that the encapsulation process does not induce significant conformational changes. Therefore, these FTIR results suggest that the formulation strategy employed is suitable for protein delivery applications, as it does not promote insulin denaturation during nanoparticle synthesis, in agreement with previous reports [33].

The fluorescence emission profiles obtained for tyrosine and tryptophan provide complementary evidence of the structural conformation of insulin after encapsulation within chitosan–alginate nanoparticles. Intrinsic fluorescence spectroscopy demonstrates that the formulation strategy maintains the conformational structure of insulin.

The hydrodynamic diameter obtained for released insulin (≈1.145 nm) is indicative of a small, monomeric protein species. Although previous DLS studies typically report insulin hydrodynamic diameters in the range of 1.8–2.7 nm in aqueous solution [44], the most relevant observation in the present analysis is the absence of larger size populations. The lack of detectable peaks above 3 nm confirms that the released insulin does not undergo significant aggregation or structural destabilization during the encapsulation, release, or measurement processes. Aggregation would be expected to manifest as additional populations at higher hydrodynamic diameters, which are not observed in this case. These results corroborate that the signal monitored during the release experiments corresponds to insulin in a non-aggregated state. Together with the FTIR and intrinsic fluorescence analyses, the DLS data reinforce the effectiveness of the chitosan–alginate nanoparticulate system in protecting insulin during encapsulation and enabling its release without inducing denaturation.

The broad diffraction halo observed in all samples confirms that the chitosan–alginate nanoparticulate system is predominantly amorphous, consistent with the formation of a polyelectrolyte complex mediated by ionic crosslinking with TPP. The absence of characteristic crystalline peaks of insulin in recombinant human insulin-loaded nanoparticles suggests that the protein is not present in a crystalline state but rather is molecularly dispersed or exists in an amorphous form within the polysaccharide matrix. This amorphous physical state is advantageous for therapeutic applications, as drug amorphization is commonly associated with enhanced solubility and improved release profiles. Moreover, avoiding a crystalline protein network reduces the energetic barrier associated with crystal lattice disruption during dissolution, facilitating insulin release in physiological media [45].

Thermogravimetric analysis (Figure 16 and Figure 17) was used to evaluate the thermal stability of the system and provide indirect evidence of insulin incorporation within the chitosan–alginate nanoparticles. All samples exhibited an initial mass loss below 150 °C, attributed to the removal of physically adsorbed water [46], with higher losses observed for the polymeric systems compared to pure insulin, reflecting differences in hydration and polymer–water interactions. In the main degradation region (200–400 °C), blank nanoparticles showed a single decomposition event associated with polysaccharide degradation [47], while pure insulin exhibited a sharper thermal breakdown profile. In contrast, insulin-loaded nanoparticles displayed a broader, multi-step degradation pattern, indicating altered thermal stability due to protein–polymer interactions. The reduced residual mass at high temperatures in the loaded system compared to the blank further supports successful insulin incorporation. Changes in thermal behavior confirm the effective encapsulation of insulin and its interaction with the chitosan–alginate matrix.

## 5. Conclusions

This study developed and characterized a chitosan–alginate nanoparticle system for the encapsulation and pH-responsive release behavior of recombinant human insulin, which was synthesized by ionic gelation under aqueous conditions. The formulation exhibited favorable physicochemical properties, supporting efficient electrostatic complexation between insulin and the polysaccharide matrix.

Comprehensive structural, spectroscopic, and morphological analyses suggest that insulin retains its characteristic chemical features after encapsulation and remains molecularly dispersed within an amorphous polymeric network. However, these results represent indirect evidence and should be interpreted as indicative of structural preservation rather than definitive proof of complete biological integrity.

Release studies revealed a pH-dependent delivery profile governed predominantly by diffusion-controlled mechanisms, with rapid release under acidic conditions and sustained release at physiological pH. Overall, the present work provides a formulation-level and physicochemical proof-of-concept for a chitosan–alginate-based insulin delivery system.

It is concluded that chitosan–alginate nanoparticles represent a biocompatible, scalable, and environmentally safe alternative for the release of insulin. The lyophilized matrix further enhances practicality by allowing storage and transport without refrigeration, offering a cost-effective alternative particularly suited to resource-limited settings.

## Figures and Tables

**Table 1:** pH values recorded during the optimized synthesis of chitosan–alginate nanoparticles loaded with insulin.

Solution	pH
NaOH (0.01 M)	10
HCl (0.01 M)	2
Insulin *	5.4
Acetic acid (0.4 M)	3
TPP + NaOH	11
ALG + NaOH	11
Insulin + HCl	2
CS + acetic acid	3
Insulin + HCl + NaOH + TPP	4
Insulin + HCl + NaOH + TPP + ALG	4
Final colloid	4

* Recombinant Human Insulin.

**Table 2:** Physical characterization of chitosan–alginate nanoparticles loaded with insulin (0.25 mg/mL) and blank nanoparticles. Stability at 4 °C after 2 days of storage. Abbreviations: PDI: polydispersity index; LC%: loading capacity; EE%: encapsulation efficiency.

	Size Distribution by Intensity (nm)	Size Distribution by Number (nm)	PDI	Zeta Potential (ξ)	LC%	EE%
Blank nanoparticles	688 ± 54	82 ± 15	0.53 ± 0.02	27.5 ± 2.2	-	-
Chitosan–alginate nanoparticles loaded with insulin	663 ± 33	101 ± 20	0.28 ± 0.02	41 ± 1.7	6.7 ± 0.3	30 ± 2.6

**Table 3:** Kinetic modeling parameters for insulin release.

pH	Model	Parameter	Value	R^2^	Interpretation
4.5	Korsmeyer–Peppas	*n*	0.2895	0.9907	Fickian diffusion
4.5	Higuchi	kH	7.8055	0.9953	Diffusion-controlled release
7.4	Korsmeyer–Peppas	*n*	0.4019	0.9725	Fickian diffusion
7.4	Higuchi	kH	3.4555	0.9413	Diffusion with reduced linearity

**Table 4:** Comparison of representative insulin-loaded polysaccharide nanoparticle systems reported in the literature.

System	Particle Size	EE%	Release Behavior	Biological Validation	Ref.
Chitosan and Arabic gum nanoparticles	150–200 nm	25–30	Burst effect of insulin (30% to 50% release) in the initial 5 to 15 min at pH 1.2, sustained release profiles observed at pH 6.5 and 7.2.	In vitro	[39]
Chitosan nanoparticles	318 nm	99	Initial rapid release of approximately 30% in 1 h (pH 2.5), sustained release profiles observed at pH 6.6 and 7.0.	In vitro	[1]
Alginate-dextran sulfate microspheres	68–108 µm	86–94	Initial burst effect at pH 1.2, with nearly 80% released.	In vitro	[40]
Alginate-chitosan microspheres	7.5 µm	56	32% of insulin released during a simulated gastric transit time of 2 h.	In vitro and in vivo	[41]
Chitosan–alginate nanoparticles	663 ± 33 nm by intensity distribution and 101 ± 20 nm by number distribution	30 ± 2.6	Insulin exhibited a rapid release at pH 4.5, reaching completion within 1 h, whereas a sustained release profile was observed at pH 7.4 (86.46% at 72 h).	In vitro	This work

## Data Availability

Data are contained within the article.

## Abbreviations

The following abbreviations are used in this manuscript:

ALG	Sodium alginate
ATR	Attenuated Total Reflectance
CS	Chitosan
DLS	Dynamic Light Scattering
EE%	Encapsulation Efficiency
FTIR	Fourier Transform Infrared Spectroscopy
HCl	Hydrochloric Acid
NaOH	Sodium Hydroxide
nm	Nanometer
PDI	Polydispersity Index
pH	Potential of Hydrogen
PO_3_	Phosphate Group (Trivalent)
SEM	Scanning Electron Microscopy
TPP	Sodium Tripolyphosphate
Tyr	Tyrosine
Trp	Tryptophan
UV–Vis	Ultraviolet–Visible Spectroscopy
α-helix	Alpha-helix
β-sheet	Beta-sheet
ξ	Zeta Potential
